# Wenshen Yangxue decoction improves endometrial receptivity recovery and promotes endometrial angiogenesis in a rat model

**DOI:** 10.1080/13880209.2018.1510973

**Published:** 2018-12-11

**Authors:** Mingwei Xin, Junqin He, Wei Yang, Xiaodan Yin, Jingshang Wang

**Affiliations:** Department of Traditional Chinese Medicine, Beijing Obstetrics and Gynecology Hospital, Capital Medical University, Beijing, PR China

**Keywords:** Microvessel density, VEGF, HIF-1α, PI3K

## Abstract

**Context:** Wenshen Yangxue decoction (WSYXD) is a famous traditional Chinese medicine (TCM) formula and has been used in infertility treatment, but the exact mechanism is still unknown.

**Objectives:** To determine if WSYXD improves endometrial receptivity recovery and promotes endometrial angiogenesis in a rat model.

**Materials and methods:** A total of 100 proestrus female SPF Wistar rats were randomly assigned into five groups: control (saline), model (saline and hydroxyurea solution), high (5.2/100 g), middle (2.6/100 g) and low (1.3/100 g) WSYXD dose groups for 10 d. The microvessel densities, endometrial microstructure, as well as blastocysts number, were observed, followed by detection of angiogenesis-related gene/protein expression by immunohistochemistry, western blot and quantitative real-time polymerase chain reaction (RT-PCR), respectively.

**Results**: Compared with the model group, the blastocyst number in WSYXD middle and high groups were significantly increased (4.50 ± 3.11 vs. 13.00 ± 2.12, 14.00 ± 1.83, *p* < 0.01). Lower MVD can be found in the model group (4.7) when compared with the normal control (13.7), middle (8.4) and high (9.7) dose groups. Additionally, significant differences were observed in VEGF, HIF-1α, p-AKT, p-PI3K, Ang1 and Ang2 (all *p* < 0.01) among different groups.

**Discussion and conclusions:** In conclusion, WSYXD could help endometrial receptivity recovery and promote endometrial angiogenesis through PI3K, HIF-1α signalling and VEGF expression regulation. This study provides molecular evidence for application of WSYXD in the clinic and promotes new drug development from TCM.

## Introduction

Worldwide, 48.5 million couples suffered from infertility-related matters in 2010 (Mascarenhas et al. 2012), and the incidence of infertility is still increasing. Researchers have identified impaired endometrial receptivity as the major reason for this low fertility (Gong et al. 2013). Endometrial receptivity is defined as a transient period, during which the endometrial epithelium allows implantation, which is also known as the window of implantation (WOI) (Mathyk et al. 2017). Actually, endometrial receptivity is a very complex process involving a good quality embryo, a receptive endometrium and the synchronisation between the developmental stages of the embryo itself.

Several factors have been reported to be associated with endometrial receptivity. For instance, prolonged embryo culture is associated with greater uterine receptivity, while cryopreserved embryos are associated with poorer uterine receptivity (Roberts et al. 2016). Progesterone is considered to be essential for endometrial receptivity in primates (Ghosh and Sengupta 2014). MicroRNAs miR-30b, miR-30d and miR-494 can regulate human endometrial receptivity (Altmae et al. 2017). Numerous genes are also believed to act as positive or negative regulators of endometrial receptivity, such as leukaemia inhibitory factor, glutathione peroxidase 3, interleukin-15 and mucin1 (Ghosh and Sengupta 2014). HRG C633T has an impact on various aspects of fertility including endometrial receptivity (Mathyk et al. 2017). Recently, studies have focused on angiogenesis which, together with increased vascular permeability, are considered to be the key factors in endometrial decidualisation, embryo implantation and placentation (Carlone and Rider 1993; Lindgren et al. 2016).

As a kind of cytokine, angiopoietin (Ang) has been proved to promote angiogenesis (Metheny-Barlow and Li 2003). Ang-1 concentration peaked in the mid-secretory phase, while Ang-2 significantly increased in the late secretory phase. Immunohistochemical analysis showed that Ang-1 existed mainly in the perivascular matrix, indicating that Ang-1 promoted angiogenesis by playing roles on the subepithelial capillary plexus (Lee et al. 2008). Ang-2 over-expression disrupted blood vessel formation, resulting in implantation failure in transgenic mice (Maisonpierre et al. 1997).

Like Ang, vascular endothelial growth factor (VEGF) is essential for normal vascular development in mice. The expression peak of VEGF was in consistence with that of microvessel density (MVD), suggesting that VEGF play a role in promoting angiogenesis (Raine-Fenning 2008). Clinically, a relative hypoxia environment (2 ∼ 3% O_2_) was found to be necessary for embryo implantation (Jauniaux et al. 2000). In addition, hypoxia-inducible factor 1alpha (HIF-1α) is highly expressed in endometrial glandular epithelium in pregnant women, and there is a trend of expression increasing as time passes (Dubinsky et al. 2010). HIF-1α and HIF-2α can stimulate angiogenesis by activating of VEGF in tumour cells, and expression of HIF-1α was associated with VEGF pathway up-regulation as well as increased standard MVD (sMVD) (Sivridis et al. 2002). All of which suggested that HIF-1α and VEGF play important roles in endometrial angiogenesis.

PI3K/AKT signal pathway is the regulatory centre for angiogenesis. Previous studies reported that PI3K/AKT pathway was associated with HIF-lα synthesis and HIF-1α dependent VEGF transcription (Choi et al. 2010; Altmae et al. 2013; Bidarimath et al. 2014). C-reactive protein can up-regulate VEGF expression, promoting angiogenesis by activating HIF-1α *via* PI3K pathway (Gamundi-Segura et al. 2015; Chen et al. 2016). This study involved VEGF, HIF-1α, PI3K/AKT pathway and angiogenesis, suggesting that there may be a potential relationship which should be investigated more detailed.

WSYXD is a famous traditional Chinese medicine (TCM) formula, which combines warming kidney-yang herbs (such as *Epimedii Folium*, *Cistanches Herba*) with high dose blood-activating medicines (such as *Paeoniae Radix Rubra*, *Typhae Pollen*). A large number of clinical trials have verified the significant efects of WSYXD on the infertility treatment. For example, WSYXD had a positive effect of on angiogenesis in the endometrium clinically (Xin et al. 2011, 2012). However, the exact mechanism remains unknown. Given that WSYXD contains high dose of blood-activating promoting blood circulation maybe better herbs, which are rich sources of compounds that may associate with angiogenesis (Yu et al. 2012). In this study, we hypothesized that WSYXD may alter endometrial blood flow by regulating endometrial angiogenesis, leading to improvement of endometrial receptivity.

In this study, the MVD endometrial microstructure and the number of the blastocysts were initially observed to investigate effects of WSYXD on the endometrium in rat model. Then we detected the expressions of several angiogenesis-related genes/proteins by immunohistochemistry, western blot or quantitative real-time polymerase chain reaction (RT-PCR) to explore the impact of WSYXD on these pathways. These results may provide molecular evidence for application of WSYXD in the clinic and promote new drug development from TCM.

## Materials and methods

### Preparation of WSYXD

The drugs present in WSYXD were obtained from the Capital Medical University (Beijing, China) which has good effect clinically (Xin et al. 2011, 2012, 2016). An aqueous extract of WSYXD was prepared in accordance with the following procedure. WSYXD contains 23 herbal materials, and the ratio is presented in Table 1. Herb pieces of *Cistanches Herba* (10 g), *Cervi Cornu* (15 g), *Epimedii Folium* (10 g), *Cuscutae Semen* (10 g), *Lycii Fructus* (10 g), *Rubi Fructus* (10 g), *Lycopi Herba* (10 g), *Leonuri Herba* (12 g), *Achyranthis Bidentatae Radix* (10 g), *Spatholobi Caulis* (15 g), *Carthami Flos* (10 g), *Salviae miltiorrhizae Radix et rhizoma* (10 g), *Angelicae sinensis Radix* (10 g), *Typhae Pollen* (10 g), *Bupleuri Radix* (6 g), *Cyperi Rhizoma* (10 g), *Aucklandiae Radix* (5 g), *Paeoniae Radix Rubra* (10 g), *Chuanxiong Rhizoma* (10 g), *Rehmanniae Radix Praeparata* (10 g), *Corni Fructus* (10 g), *Notopterygii Rhizoma et Radix* (6 g) and *Asari Radix et Rhizoma* (3 g) were purchased from Beijing Tong-Ren-Tang Chinese Medicine Co. Ltd. (Beijing, China) in October 2014, and authenticated by Prof. Songquan Zhao. The components were mixed in proportion and were macerated for 5 h with 10 volumes of distilled water and then decocted for 2 h. The cooled extract was filtered. The extraction procedure was repeated twice. The extracts were then combined and concentrated by boiling to a final volume of 100 mL (4.12 g/mL). This dilution was used in the following preliminary experiments in a range of concentrations (between 1.3 and 5.2 g/100 g).

**Table 1.** The composition of WSYXD.

Component			Ratio (%)
Latin name	Species	Family	
Cistanches Herba	*Cistanche deserticoLa* Y. C. Ma	*Orobanchaceae*	4.5
Cervi Cornu	*Cervus elaphus* Linnaeus	*Bovidae*	6.7
Epimedii Folium	*Epimedium brevicornu* Maxim.	*Berberidaceae*	4.5
Cuscutae Semen	*Cuscuta australis* R. Br.	*Convolvulaceae*	4.5
Lycii Fructus	*Lycium barbarum* L.	*Solanaceae*	4.5
Rubi Fructus	*Rubus chingii* Hu	*Rosaceae*	4.5
Lycopi Herba	*Lycopus lucidus* Turcz. var. *hirtus* Regel	*Labiatae*	4.5
Leonuri Herba	*Leonurus j a ponicas* Houtt.	*Labiatae*	5.4
Achyranthis bidentatae Radix	*Achyranthes bidentata* B1.	*Amaranthaceae*	4.5
Spatholobi Caulis	*Spatholobus suberectus* Dunn	*Leguminosae*	6.7
Carthami Flos	*Carthamus tinctorius* L.	*Compositae*	4.5
Salviae miltiorrhizae Radix et rhizoma	*Salvia miltiorrhiza* Bge.	*Labiatae*	4.5
Angelicae sinensis Radix	*Angelica sinensis* (Oliv.) Diels	*Apiaceae*	4.5
Typhae Pollen	*Typha angustifolia* L.	*Typhaceae*	4.5
Bupleuri Radix	*Bupleurum chinense DC.*	*Apiaceae*	2.7
Cyperi Rhizoma	*Cyperus rotundus L.*	*Cyperaceae*	4.5
Aucklandiae Radix	*Aucklandia lappa* Decne	*Compositae*	2.7
Paeoniae Radix Rubra	*Paeonia lactiflora* Pall.	*Ranunculaceae*	4.5
Chuanxiong Rhizoma	*Ligusticum chuanxiong* Hort	*Apiaceae*	4.5
Rehmanniae Radix Praeparata	*Rehmannia glutinosa* Libosch.	*Scrophulariaceae*	4.5
Corni Fructus	*Cornus officinalis* Sieb.et Zucc.	*Cornaceae*	4.5
Notopterygii Rhizoma et Radix	*Notopterygium incisum* Ting ex H. T. Chang	*Apiaceae*	2.7
Asari Radix et Rhizoma	*Asarum heterotro poides* Fr.Schmidt var.*mandshuricum* (Maxim.) Kitag.	*Aristolochiaceae*	1.3

### HPLC analysis of WSYXD

WSYXD extract was dissolved in 30% ethanol to obtain appropriate concentration for HPLC analysis. The identification of chemical constituents in WSYXD extract was carried out on an Agilent Technologies 1200 system (Agilent Crop., MA, USA), and performed with a TC-C18 column (4.6 mm × 250 mm, 5 μm). The mobile phase was composed of acetonitrile (solvent A) and 0.1% phosphoric acid (solvent B): 0–20 min (0–10%, A), 20–40 min (10–35%, A), 40–60 min (35–70%, A). The injection volume of sample was 20 μL, and the testing time was 60 min; in addition, the flow rate was 1 mL/min, the column temperature was set at 30 °C and the detection wavelength was set at 327 nm in 0–15 min, 316 nm in 16–30 min, 286 nm in 31–60 min, to detect several components in WSYXD. The chlorogenic acid, hydroxysafflor yellow A, ferulic acid and salvianolic acid were mixed together as standard control.

### Animal model generation and treatment

In total, 100 proestrus female as well as 50 male SPF Wistar rats (200–220 g) were purchased from Beijing Vital River Laboratory Animal Technology Co., Ltd. The female rats were randomly divided into five groups of 20 rats each: control, model, high, middle and low dose groups. Vaginal smears were collected at 9 am every day and modelling started once static phase identified (plenty of white blood cells, or with a little flat epithelial cells but no keratinocyte in vaginal smear). For the control group, only saline (1 mL/100 g body weight) was given by gavage for 10 d. The model group received intragastric administration of saline (0.5 mL) in the morning and hydroxyurea solution (450 mg/kg/d) in the afternoon for 10 d. While for the other groups, saline was replaced by WSYXD at different doses (low dose – 1.3/100 g; middle dose – 2.6/100 g; and high dose – 5.2/100 g). In addition, the model and WSYXD group were given adrenaline (0.3 mg/kg/d) by subcutaneous injection from the fourth day to the tenth day. After model generation, the female rats were then placed with males in the ratio of 2:1 every 6 pm. The day on which vaginal plug was identified was considered the first day of pregnancy (P1). Then 10 randomly chosen females were sacrificed on P5, followed by endometria collection. These tissues were divided into four parts: one for MVD determination, three for gene expression analysis using immunohistochemistry, RT-PCR and western blot, respectively. On P8 the remaining rats were killed. The whole uteri were collected for blastocyst number and SEM examination. Saline or WSYXD was continuously administrated to each group until they were sacrificed. These experiments were approved by the Animal and Human Ethics Board of Beijing Obstetrics and Gynaecology Hospital, Capital Medical University and conducted in accordance with Animal Research Committee Guidelines.

### Scanning electron microscope (SEM) and implantation examination

On P8, 10 rats from each group were sacrificed. After removing fat and connective tissue, the uteri were separated and the conceptuses were removed. The number of implantation was recorded. The implantation rate was calculated as the number of implantation/10 rats.

The medial ⅓ right uteri were selected. Fixation was performed by using 2.5% glutaraldehyde, followed by PBS washing, serial acetone dehydration and soaking in isoamyl acetate for 3 h. Then the tissue was critical point-dried, silver conductive adhesive and metallic film was used for the tissue coating. Then SEM and image capture were performed.

### MVD determination by lectin immunohistochemistry

The MVD was calculated as the number of lectin-positive vessels. For each section, six random fields at 400x magnification were selected. The microvessels were marked by CD31-positive endothelial cell, and were quantified using Diagnostic Instruments Spot-II digital software (Diagnostic Instruments, Inc., Sterling Heights, MI). Single stained cell or cell cluster was also quantified as one vessel, but vessels with thick muscle layer or large diameter (larger than the size of eight red cells) were excluded. Then the average number was treated as MVD.

### VEGF expression analysis with immunohistochemistry and RT-PCR

After paraffin fixation, the tissues were sectioned into 4 μm thickness slides, followed by dewaxing, rehydration and blocking. Then primary antibodies against VEGF (ab1316, 1:50, Abcam, Cambridge, UK,) were used for immunolabelling. And the slides were incubated with secondary antibodies at RT for 30 min, followed by incubation with a diaminobenzidine tetrahydrochloride (DAB) substrate kit (ZLI-9017, Zhong Shan Jin Qiao, Beijing, China). The negative controls were treated with the same procedure except with the PBS during the primary antibody incubation step. VEGF expression was evaluated at 400× magnification (0.24 mm^2^/field). A digital camera (Motic BA400, Xiamen, China) was used for image capture. Five fields were randomly selected on each section to obtain an average optical density.

Total RNA from endometrium of rats was extracted by using Trizol reagent (Invitrogen, Carlsbad, CA) according to the manufacturer’s instructions. Complementary DNA (cDNA) was obtained by using a PrimeScript first Strand cDNA Synthesis Kit (Takara, Dalian, China), followed by total amplification using one-step SYBR Green I PCR Master Mix (Takara) with following conditions: 30 s of polymerase activation at 95 °C, 40 cycles of 95 °C for 5 s and 60 °C for 34 s. The primers used for PCR amplifications were: 5′-ACGACAGAAGGGGAGCAGAA-3′ and 5′-GCACTCCAGGGCTTCATCAT-3′. GAPDH was selected as an internal control gene, and the primers used GAPDH was 5′-CCTGCACCACCAACTGCTTAACTTCAACAGCGACACCCACT-3′ (forward) and 5′-ATGACCTTGCCCACAGCCTGCCAAATTCGTTGTCATACCAG-3′ (reverse). The comparative method (2^−ΔΔCt^) was employed to quantify gene expression.

### Western blot analysis

Rat endometrium tissues were initially homogenized and lysed in radio-immunoprecipitation assay (RIPA) buffer (Beijing Dingguo Changsheng Biotechnology Co. Ltd., China) supplemented with proteinase inhibitor (Lot#4693116001, Roche). Lysates were then centrifuged for 20 min at 12,000 *g*. The bicinchoninic acid (BCA) (Beijing Dingguo Changsheng Biotechnology Co. Ltd., China) was used for quantification of protein concentration. Equivalent amounts of protein (20 μg) were then used for western blot with primary antibodies of VEGF (ab53465, Abcam, UK), PI3K (ab191606, Abcam, UK), P-PI3K (ab182651, Abcam, UK), AKT (ab8805, Abcam, UK), P-AKT (ab38449, Abcam, UK), Ang-1 (ab102015, Abcam, UK), Ang-2 (ab155106, Abcam, UK) and HIF-1a (ab1, Abcam, UK) at 4 °C with gentle shaking overnight. After washing with TBS-T for three times, the membranes were then incubated with the secondary antibody at RT for 1 h and detected using an ECL plus kit (Beijing Dingguo Changsheng Biotechnology Co. Ltd., China). The ECL signals were detected with Quantity One software (Bio-Rad, Hercules, CA) and blot intensities were quantified by using imageJ (NIH, Bethesda, MD) software. Tubulin (ab8245, Abcam, UK) was used as an internal control to validate the amount of protein loaded onto the gels.

### Statistical analysis

All the continuous variables were expressed as mean ± standard deviation (SD). One-way ANOVA was performed to detect difference among the five groups. Student’s *t*-test was used for the difference analysis. A *P* value of more than 0.05 was considered as statistical significance. SPSS version13.0 for Windows (SPSS, Armonk, NY) software was used for all the statistical analyses.

## Results

### Identification of main chemical compositions of WSYXD

**Figure 1.**
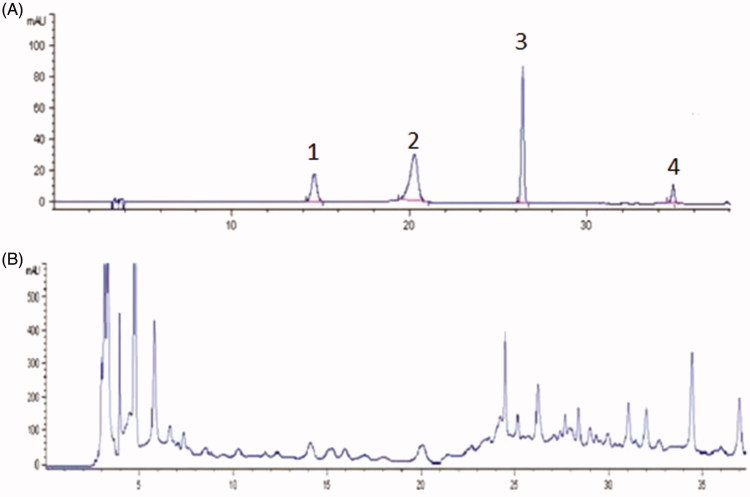
Identification of the main chemical composition of WSYXD. A. Peak 1, chlorogenic acid; Peak 2, hydroxysafflor yellow A; Peak 3, ferulic acid; Peak 4, salvianolic acid. B. The spectrum of WSYXD on HPLC.

The chlorogenic acid, hydroxysafflor yellow A, ferulic acid and salvianolic acid were identified from WSYXD by using HPLC assay with references compounds (Figure 1). Our results indicate that these four compounds are important constituents of WSYXD. The content of the chlorogenic acid was 0.384 mg/g, the hydroxysafflor yellow A was 0.0369 mg/g, the ferulic acid was 0.1506 mg/g, and the salvianolic acid was 0.206 mg/g.

### WSYXD improves endometrial receptivity

**Figure 2.**
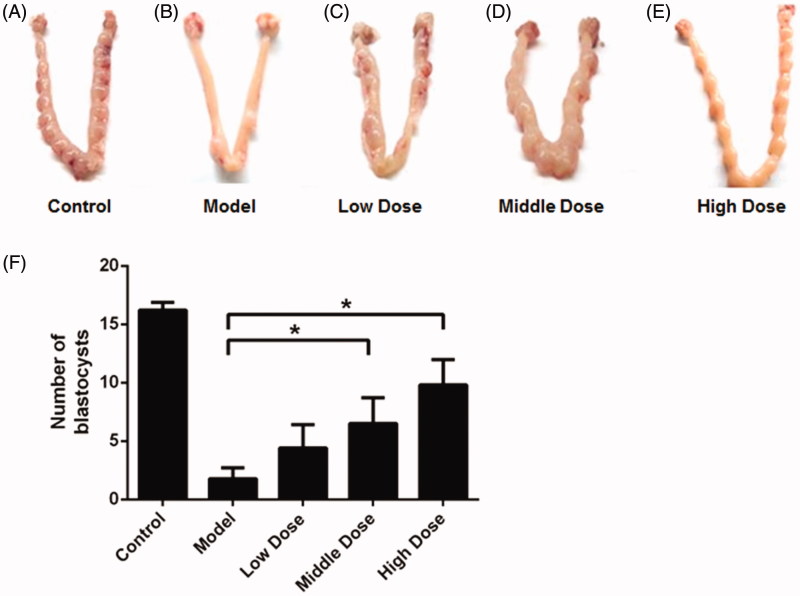
The conceptuses in different groups. A. control group; B. model group; C. low dose group; D. middle dose group; E. high dose group; F. the comparison of blastocyst number among five groups. *p< 0.01.

As shown in Figure 2, the number of blastocysts in control group was 16.20 ± 2.15 (implantation rate = 100%). While it was 4.50 ± 3.11 (40%) in the model group, 11.00 ± 4.97 (40%), 13.00 ± 2.12 (50%), and 14.00 ± 1.83 (70%) in the low, middle and high dose group, respectively. Significant differences in blastocyst number can be found between model and WSYXD administration groups (*p* < 0.01, Kruskal–Wallis statistic = 23). While in implantation rate, there was no significant difference between model and low dose group.

**Figure 3.**
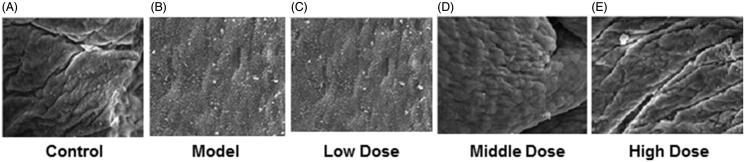
Endometrial surface detected by SEM. A. control group; B. model group; C. low dose group; D. middle dose group; E. high dose group.

Meanwhile, there were a large number of pinopodes but little short microvilli on the endometrial surface in control and high dose groups (Figure 3(A,E)). However, no pinopode but numerous microvilli can be found in the model and low dose groups (Figure 3(B–C)). For middle dose group, pinopodes existed in only parts of endometrium (Figure 3(D)).

### WSYXD increases MVD in a rat model

**Figure 4.**
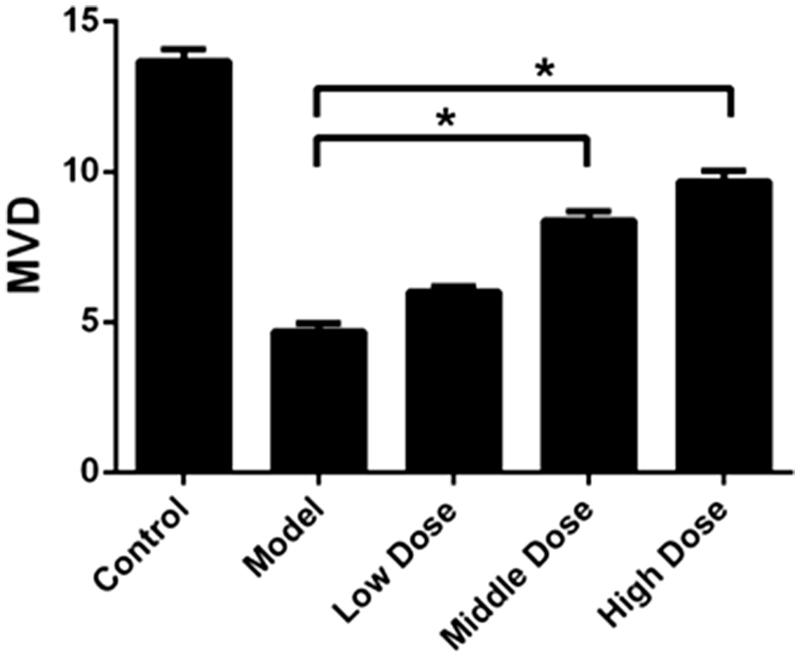
Comparisons of MVD among five groups. *p< 0.01.

As shown in Figure 4, The model group showed significantly lower MVD when compared with the control, middle and high dose groups (*p* < 0.001, F = 140, *R*^2^ = 0.92). The average MVD of model group was only 4.7, while the control group was 13.7. It is interesting that the WSYXD can increase the MVD significantly: the average MVD of low, middle and high dose group were 6.0, 8.4 and 9.7, respectively.

### Increasing of VEGF gene expression upon WSYXD treatment

**Figure 5.**
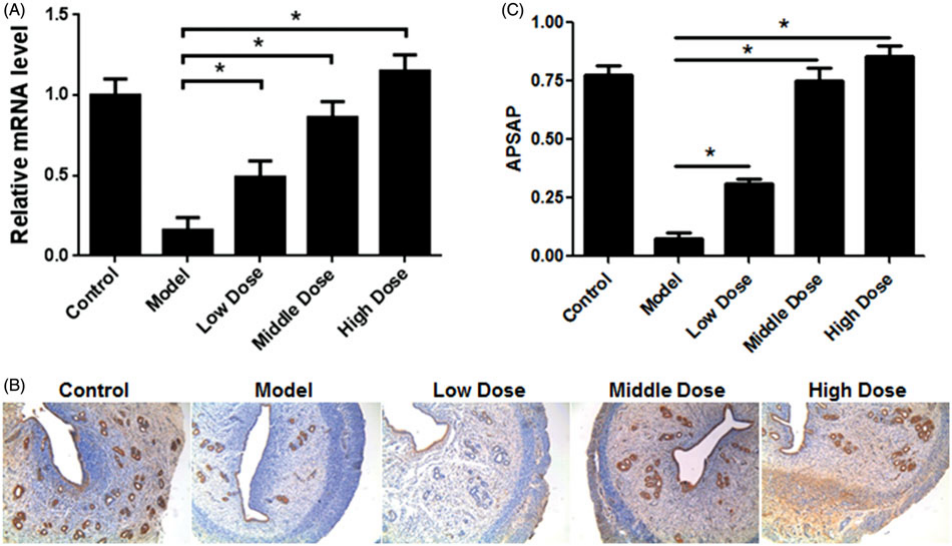
RT-PCR and IHC analysis of VEGF in different groups. A. RT-PCR results of VEGF in five groups. B. The representative results of VEGF in five groups by IHC. C. The analysis of VEGF expression levels by Image J based on ten photos in each group. ASASP, Average positive staining area percentage. *p< 0.01.

Quantitative RT-PCR analysis showed that overall relative quantity of VEGF mRNA was significantly lower in the model group than those in the control and WSYXD administration groups (Figure 5(A)). The change in model group was 0.16-fold; while in the low, middle, and high dose groups, it was 0.49-, 0.86-, and 1.15-fold, respectively (Figure 5(A)). Moreover, the significant difference in mRNA and protein expression level of VEGF expressions were observed by qRT-PCR and immunohistochemistry assays (Figure 5(B–C)).

**Figure 6.**
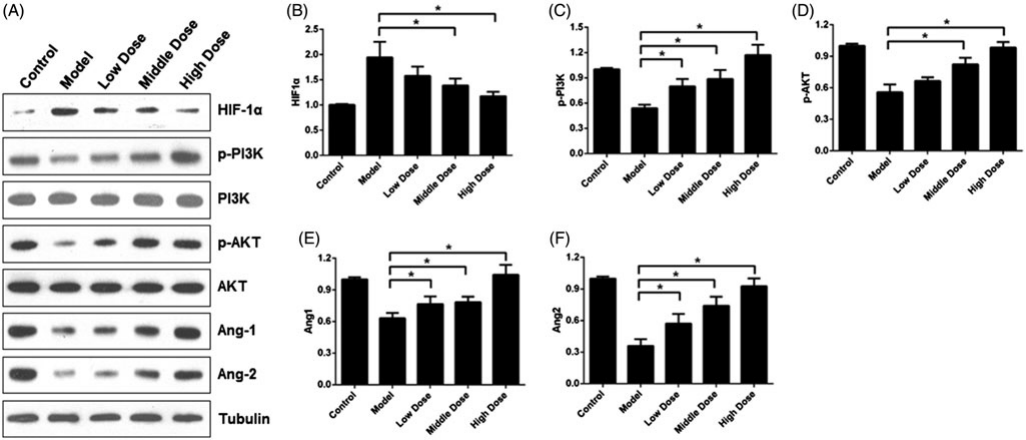
Western blot analysis of rat endometrium tissues. A. WB results for HIF-1α, p-PI3K, p-AKT, Ang1 and Ang2. B-F. The histograms of western blot analysis for HIF-1α (B), p-PI3K (C), p-AKT (D), Ang1 (E) and Ang2 (F) in the five groups. *p< 0.01.

### PI3K/AKT signal pathway was associated with WSYXD efficacy

Compared with model group, significantly different expression of HIF-1α and p-AKT can be observed in control, middle and high dose groups (*p* = 0.009, *F* = 4.3, *R*^2^ = 0.41 for HIF-1α, Figure 6(B); *p* < 0.001, *F* = 14, *R*^2^ = 0.69 for p-AKT, Figure 6(D)). Furthermore, the model group the showed significantly higher expression, while for p-AKT it was significantly lower. There was no significant difference between model and low dose groups in HIF-1α and p-AKT. Moreover, no significant difference existed among these groups in PI3K and AKT. The model group expressed significantly lower p-PI3K, Ang1 and Ang2 when compared with the control, low, middle and high dose groups (*p* < 0.001, *F* = 7.75, *R*^2^ = 0.55 for p-PI3K, Figure 6(C); *p* < 0.001, *F* = 7.84, *R*^2^ = 0.5564 for Ang1, Figure 6(E); *p* < 0.001, *F* = 13.47, *R*^2^ = 0.68 for Ang2, Figure 6(F)).

## Discussion

In TCM, endometrial receptivity is considered as female reproduction ability, which depends on the balance among Kidney-Qi, Tiangui, Chongren, and the Baogong (uterus). According to TCM theory, kidneys reproductions, sufficient Kidney-Qi is the essence while blood and Qi is the material basis of reproduction. The ‘golden mirror of Medicine’ suggested that damage in Chongren resulted in infertility. ‘Fu Qingzhu Nüke’ also believed that normal fertility should regulate menstruation firstly, sufficient blood would help the uterus receive sperm. Thus, blood is important for women according to TCM theory and once blood is sufficient, ChongRen channels are not blocked; it will be easy for women to have the fertility ability. Sufficient Kidney-Qi and Chongren cooperation are the basic conditions of fertility, which can promote follicular development and endometrial decidualisation. In TCM, the principles for infertility treatment are tonifying the kidney essence and regulating the balance between body Qi and blood. Clinically, we targeted the basic pathogenesis by warming kidney and nourishing blood to treat the presentations. Kidney is the source of essence and blood. Warming kidney could help essence and blood production. Our drug WSYXD can supplement Kidney-Qi, sufficient Kidney-Qi results in blood production. In addition, WSYXD contains blood-activating herbs, which may regulate Qi movement and remove blood stasis in bodies, then enhancing WSYXD effect to improve the blood perfusion in peri-uterus areas and providing a favourable environment for embryo implantation.

As an index of evaluation of angiogenesis, higher MVD stands for abundant capillary. In our study, the control, middle and high dose groups showed higher MVD when compared with the model group, which were in consistence with VEGF expression evaluated by immunohistochemistry and western blot analysis, especially for Ang1 and Ang2. Moreover, the number of MVD was positively correlated with that of pinopodes. In the model group, normal angiogenesis was broken because of the use of hydroxyurea, which in return resulted in insufficient blood supply of endometrium and reduced MVD as well as pinopodes. While in the middle and high dose groups, WSYXD restored regular angiogenesis in endometrium, then high expression of pinopodes could be found in the highly vascularized area (with increased MVD). These results indicated that poor angiogenesis and insufficient pinopodes may contribute to the decrease of uterine receptivity, causing implantation failure. WSYXD has the ability of promoting endometrial angiogenesis and may help endometrial receptivity recovery.

Pinopodes are flower-shaped protrusions, which can be found on apical epithelial cells in the endometrium of the uterus (Adams et al. 2001). Currently, debates still exist concerning the usefulness of pinopode as a marker for endometrial receptivity (Quinn and Casper 2009). Cortínez et al. (2005) considered that pinopode can be used as a morphological marker for endometrial receptivity. Sudoma et al. (2011) believed that pinopodes were associated with endometrial receptivity, while Quinn et al. (2007) deemed that pinopode cannot predict endometrial ‘implantation window’ and thus had no relationship with endometrial receptivity. Although debates still exist, the majority of studies have found expression of various cytokines and adhesion molecules which were related to embryo implantation on the surface of pinopodes; meanwhile, clinical applications showed that pregnancy rate increased significantly once development of pinopodes was improved. In this sense, usefulness of pinopodes as marker for endometrial receptivity should be affirmed. Our study showed that high and middle dose groups had a large number of pinopodes on the endometrial surface. In addition, the number and morphology of pinopodes showed a reverse trend to those of microvilli, suggesting that expression of pinopodes was closely related to implantation. Because the existance of pinopodes can significantly increase the accessible area of endometrium, leading to improvement of the ability of embryo adhere to the endometrium. Our results indicated that WSYXD played positive roles in endometrial receptivity.

Interestingly, our study found significantly increased expression of p-AKT and p-PI3K, but not AKT and PI3K, which indicates that activation of PI3K pathway has been involved in the endometrial receptivity. In addition, the distribution of VEGF in WSYXD groups was different from model group. Last, our results also identified the involvement of HIF-1α in the endometrial receptivity recovery when treated with WSYXD.

## Conclusions

Our study showed that WSYXD could promote endometrial angiogenesis and help endometrial receptivity recovery *via* PI3K and HIF-1α signaling as well as VEGF expression regulation.
